# Venesection in Infantile Diseases

**Published:** 1892-01-30

**Authors:** 


### The Practitioner's Mirror and Retrospect.

[*The Editor will be glad to receive offers of co-operation and contributions from members of the profession. All letters should be addressed to* THE EDITOR, THE LODGE, PORCHESTER SQUARE, LONDON, W.]

### MEDICINE.

### VENESECTION IN INFANTILE DISEASES.

The present generation of medical practitioners have even now to bear the brunt of the popular prejudice against venesection in any form, which was excited by the absurd practice of their medical forefathers bleeding for almost every affection they were called upon to treat. The majority of present-day practitioners are themselves unduly biassed against venesection, although they hardly realise the extent to which the practice was once carried. In the earlier decades of the present century a certain provincial hospital with only two hundred beds, used to expend over a hundred pounds annually in leeches alone! Of late years there have been many able advocates of venesection in puerperal eclampsia, and a few other diseases. Dr. Robert Lee* directs attention to the value of leeching in some diseases of infants. He believes that this useful remedy has been too much neglected, and remarks on the cases in which experience has proved its great benefit. In five cases where the medical advisers were of opinion that recovery could not be hoped for, and in which some difficulty was experienced in obtaining the parents' consent to a plan of treatment which presented the apparent objection that in such conditions of exhaustion it could only hasten the fatal end, the results were most successful.

In one case two children, aged three and five respectively, went to Dartmouth to spend the Christmas with relatives of their parents. It was the year of the great snowstorm, that is, ten years ago, when the intense cold was the cause of serious illness and mortality. He was met at the station by the medical adviser of the family, and informed that he had come too late to be of much assistance, as the youngest child had died a few hours before, and the elder, in his opinion, could not live through the night.

The case presented the usual signs and symptoms of extensive consolidation of the lung. There was dulness and tubular breathing over the whole of the left lung. There was doubtful crepitation at the base of the right lung. The pulse was 150, the respirations were between 60 and 70, and the temperature was 104 deg. All the usual remedies had been administered, and the parents had given up hopes of the child's recovery. In the other four cases the conditions were generally similar to those above described, and the same hopeless view was entertained on the question of recovery. The children were all under six years of age. They all recovered.

Dr. Lee states that in prescribing the application of leeches in such cases as was done in the cases referred to the following directions should be given. Three or four leeches, according to the age of the child, should be applied over the right or left anterior thorax, and when the leeches fall off, the hæmorrhage should not be arrested but linseed poultices or hot fomentations be applied for two or three hours to encourage bleeding. Probably, great prostration will occur and it is proper to support the patient generously with beef tea and port wine. After the warm applications the chest should be covered with wool, but the bleeding allowed to cease spontaneously. In two of the above cases the bleeding was arrested before this occurred, and in both it was necessary to repeat the application of leeches.

Dr. Lee tells us that when a child of about four years of age he recollects being treated for an attack of true croup by leeching, and his personal experience of the great relief which followed the application of the leeches, has not been without effect upon the treatment he has usually advised in true croup when the symptoms are of a very acute character.

Another class of cases in which great relief is generally obtained from leeches applied to the precordial region are those of mitral insufficiency, and congenital heart disease, when urgent cardiac and respiratory distress demand prompt attention.

Since such an authority as Dr. Lee concludes by saying that as our individual opinions on the value of venesection, or leeching, ought to depend on personal observation rather than on any theories we may entertain, and has confined his own remarks to the results of his own experience, we are hardly justified in attempting to theorise on the mode in which leeching led to such happy results. We may, however, suggest the analogy between the treatment of cases of cardiac failure referred to above by leeching and the method of direct abstraction of blood by paracentesis cardii from an over-distended and failing right ventricle. In a recent issue we referred to the treatment of pneumonia by digitalis, and the writers whose results we then quoted attributed its beneficial action to the maintenance of powerful cardiac action. Do not Dr. Lee's cases seem to show that the earlier resort to direct cardiac stimulants may sometimes prevent the extreme engorgement which, when it has reached a certain degree, can only be relieved by leeching?

